# Case Report: Ocular toxoplasmosis in a WHIM syndrome immunodeficiency patient

**DOI:** 10.12688/f1000research.16825.2

**Published:** 2019-07-17

**Authors:** David H. McDermott, Lauren E. Heusinkveld, Wadih M. Zein, H. Nida Sen, Martha M. Marquesen, Mark Parta, Sergio D. Rosenzweig, Gary A. Fahle, Michael D. Keller, Henry E. Wiley, Philip M. Murphy

**Affiliations:** 1 Laboratory of Molecular Immunology, National Institute of Allergy and Infectious Diseases, National Institutes of Health, Bethesda, MD, 20892, USA; 2 National Eye Institute, National Institutes of Health, Bethesda, Maryland, 20892, USA; 3 Laboratory of Clinical Immunology and Microbiology, National Institute Allergy and Infectious Diseases, National Institutes of Health, Bethesda, Maryland, 20892, USA; 4 Clinical Research Directorate/Clinical Monitoring Research Program, Bethesda, MD, Frederick National Laboratory for Cancer Research sponsored by the National Cancer Institute, Bethesda, Maryland, 20892, USA; 5 Department of Laboratory Medicine, Clinical Center, National Institutes of Health, Bethesda, Maryland, 20892, USA; 6 Division of Allergy & Immunology, Children’s National Medical Center, Washington, DC, 20010, USA

**Keywords:** Toxoplasma gondii, Treatment, Retinitis, Optic neuritis, Genetic Immunodeficiency, CXCR4

## Abstract

A patient with WHIM syndrome immunodeficiency presented with sudden painless right eye blindness associated with advanced retinal and optic nerve damage. *Toxoplasma gondii *was detected by PCR in vitreous fluid but not serum.  The patient was treated with pyrimethamine/sulfadiazine for 6 weeks due to evidence of active ocular inflammation and then received prophylaxis with trimethoprim-sulfamethoxazole due to his immunosuppression.  Vision did not return; however, the infection did not spread to involve other sites.  Toxoplasmosis is rare in primary immunodeficiency disorders and is the first protozoan infection reported in WHIM syndrome.

## Introduction

*Toxoplasma gondii* is an obligate intracellular protozoan with a wide host range among vertebrates, including humans ^1^. Domestic cats and their relatives, the definitive hosts of *T. gondii*, release large numbers of unsporulated oocysts in their feces, which are then ingested by secondary hosts. Major sources of infection include ingestion of contaminated water or undercooked meat and exposure to other materials contaminated with cat feces, although transmission can also occur by transplantation, blood transfusion and laboratory accidents. Human infection has been estimated to occur in ~30% of individuals worldwide based on seroprevalence studies but usually results in lifelong subclinical infection in immunocompetent individuals. Chronic infection most commonly manifests as tissue cysts in skeletal muscle, myocardium, brain and eye. Acute toxoplasmosis in immunocompetent individuals may present as a mononucleosis-like syndrome. In the setting of acquired immunodeficiency, toxoplasmosis may occur as a result of primary *T. gondii* acquisition or reactivation of latent infection and may present as systemic illness or as a localized infection. Central nervous system toxoplasmosis is a particular problem in AIDS patients and is an AIDS-defining condition. Ocular toxoplasmosis can also occur in AIDS patients and may even be the presenting manifestation ^2^. Vertical transmission of *T. gondii* is ~40% for women who become infected during pregnancy and may cause severe congenital developmental defects involving the brain, eye and other organs. In the eye, *T. gondii* may cause a progressive and recurring necrotizing retinochoroiditis and is the most common cause of infectious uveitis worldwide. Optic neuritis is a less frequent presentation that is usually associated with worse visual outcome. Ocular toxoplasmosis occurs in patients with acquired immunodeficiency but has not previously been reported in patients with primary immunodeficiency disorders.

Warts-Hypogammaglobulinemia-Infections-Myelokathexis (WHIM) syndrome is a rare primary immunodeficiency disorder caused by autosomal dominant gain-of-function mutations in the chemokine receptor CXCR4 ^3^. The primary manifestations of WHIM syndrome are cutaneous and mucosal warts, hypogammaglobulinemia, recurrent non-invasive infections, which are usually bacterial in origin and typically affect the oto-sino-pulmonary tract and skin, and myelokathexis, a term coined for retention of mature neutrophils in bone marrow. Opportunistic and life-threatening infections in WHIM syndrome patients are rare, and significant protozoan infection has not been previously reported.

## Clinical course and management

### Initial presentation and history

A 14-year-old Hispanic male with WHIM syndrome (mutation *CXCR4 ^R334X^*) from El Salvador presented with painless sudden onset right eye blindness of at least one week’s duration. There was no history of blunt or chemical trauma to the eye, recent bacterial or viral illness, or change in medication, and he reported no eye pain, periorbital swelling, eye discharge, fever, rash or headache. The past medical history was significant for Tetralogy of Fallot which had been repaired surgically. Neutropenia was diagnosed as a neonate, resulting in recurrent upper and lower respiratory tract infections complicated by bronchiectasis and tympanic membrane perforation. He received filgrastim (G-CSF, Amgen) from ages 1–3 but this was discontinued due to bone pain. At age 13, he developed dengue fever and three successive episodes of pneumonia, prompting evaluation for primary immunodeficiency. Panleukopenia was documented (ANC 90, AMC 50, ALC 1070, platelets 122,000, CD4+ T cells 365, CD19+ B cells 11 [all per microliter]). The serum IgA level was low, but IgG and IgM levels were within normal limits. Vision was normal. After obtaining informed consent on an NIH IRB-approved protocol for immunodeficiency screening (05-I-0213), genetic testing of a blood sample identified heterozygous *CXCR4 ^R334X^*, the most common WHIM syndrome genotype. The parents were unaffected. Three months later the patient experienced complete vision loss in the right eye but was otherwise asymptomatic.

### Diagnosis

**Figure 1.**
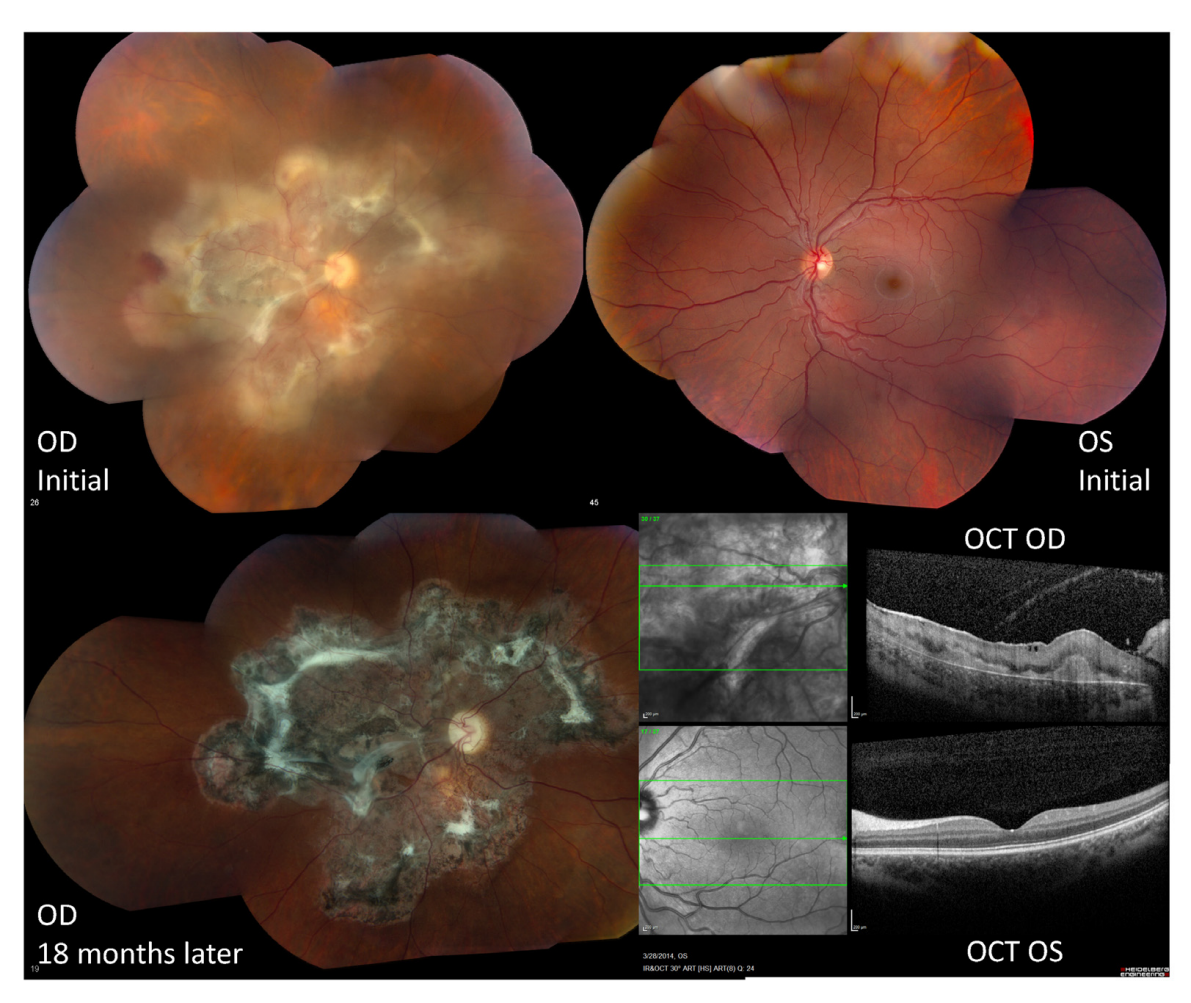
Shown on top are montage fundoscopic images of the patient’s right (OD) and left (OS) eyes at the time of presentation. The lower right panel shows the optical coherence tomography findings at presentation for OD (top) and OS (bottom).

The patient appeared well-developed but underweight (BMI = 14.5) with mild scoliosis. Splenomegaly was noted. Classic features of WHIM syndrome were present, including cutaneous warts, hypogammaglobulinemia, and severe panleukopenia ( Table 1). A bone marrow biopsy revealed myelokathexis with an elevated 4:1 myeloid:erythroid ratio. In the right eye, light perception was absent and there was an afferent pupillary defect. Dilated fundus examination showed a pale retina with widespread white subretinal infiltrates with a necrotizing appearance in some areas, patches of subretinal fibrosis, mild vitritis and a fibrotic band reaching the optical nerve head and a pale optic nerve ( Figure 1). Spectral domain optical coherence tomography images showed variable hyper-reflective infiltration of the subretinal space throughout the macula without serous subretinal fluid, with disruption of normal lamination of the macula. B-scan ultrasound showed mild vitreous opacities with presence of a posterior hyaloid membrane still adherent to the optic disc, but separated from the retina in other areas posteriorly, with presence of a vitreoschisis cavity inferiorly, without any retinal detachment, and without any definite eye wall thickening or episcleral lucency. The left eye was normal. Cranial CT scan showed mild sinusitis. Filgrastim (1 mcg/kg/day) was given resulting in increased ANC and increased vitritis the next day, suggesting the possibility of an ongoing chronic infection. Peripheral blood cultures were negative. A vitreous biopsy showed mixed inflammatory cells, and PCR testing was positive for *T. gondii* in vitreous fluid but negative in serum and bone marrow. Serum IgG for *T. gondii* was 599 international units/ml. Tests for other viral (CMV, EBV, VZV, HSV, dengue), fungal (Histoplasma, Cryptococcus), bacterial (Bartonella, Rickettsia, Legionella, Mycobacterium) and parasitic (Leishmania, Toxocara) pathogens were negative. A diagnosis of advanced ocular toxoplasmosis with ongoing active lesions was made.

**Table 1.** Hematologic characteristics of the patient upon presentation to NIH. WBC: white blood cells; RBC: red blood cells; ANC: absolute neutrophil count; ALC: absolute lymphocyte count; AMC: absolute monocyte count; NK: natural killer cells. Measured values for the cell counts are cells/µL, except as otherwise noted; values outside of the normal reference range are bolded.

*Characteristic*	*Value*	*Reference range*
WBC	**1060**	4230–9010
RBC	5.04 × 10 ^6^	4.62–6.08 × 10 ^6^
Hematocrit (%)	**39.9**	40.1–51%
Hemoglobin (g/dL)	**13.4**	13.7–17.5
Platelets	**1.4 × 10 ^5^**	1.61–3.47 × 10 ^5^
ANC	**130**	1780–5380
ALC	**880**	1320–3570
AMC	40	30–820
Eosinophils	**10**	40–540
Basophils	10	10–80
NK	213	126–729
NK-T	59	29–299
CD3+	**663**	714–2266
CD4+	**259**	359–1565
CD4/CD45RA+	**4**	102–1041
CD8+	338	178–853
CD8/CD45RA+	**10**	85–568
CD20+	**6**	59–329
IgG (mg/dL)	724	716–1711
IgM (mg/dL)	98	15–188
IgA (mg/dL)	**9**	47–249
IgE (IU/mL)	24.2	0–90

### Treatment and follow-up

The patient was treated with oral pyrimethamine (75 mg loading dose then 25 mg/day), leucovorin (7.5 mg/day), and sulfadiazine (1500 mg 2x/day) for six weeks. After treatment, chorioretinal scarring appeared stable. Serum IgG for *T. gondii* declined to 222 IU/ml 32 months later. The macula was fibrotic and atrophic without signs of active exudative lesions over 4 years follow up, during which the patient has received daily prophylactic trimethoprim/sulfamethoxazole (800 mg/160 mg). The optic nerve is atrophic in the right eye and normal in the left. Light perception continues to be absent in the right eye but left eye vision has remained normal. After completing treatment for *T. gondii*, he was enrolled in and has successfully completed a Phase 3 double blinded clinical trial (ClinicalTrials.gov Identifier NCT02231879) comparing 14 months each of twice daily plerixafor (Sanofi) and filgrastim (Amgen) treatment and is currently receiving open label filgrastim (1 mcg/kg/day). The patient has had markedly improved growth, no significant bacterial infections, no recurrence of *T. gondii* infection, and is fully active, competing at the national level in equestrian sports.

## Discussion

To our knowledge, this is the first detailed report of localized ocular toxoplasmosis in a primary immunodeficiency disorder and the first report of a protozoan infection in WHIM syndrome. In addition, optic nerve involvement as seen in our patient is rare in ocular toxoplasmosis (<5%) ^4^.

Although symptomatic toxoplasmosis occurs frequently in the setting of acquired immunodeficiency, especially HIV infection, it is rarely associated with a primary immunodeficiency disorder. Disseminated toxoplasmosis has been reported in several patients with X-linked hyper-IgM (XLHI) syndrome ^5– 9^. Neutropenia was observed in two of these patients ^5, 7^. Two patients were receiving IVIg replacement therapy at the time toxoplasmosis was diagnosed, ^5, 8^ while two others were previously undiagnosed with XLHI and had untreated hypogammaglobulinemia ^7, 9^. Of note, one patient had been taking trimethoprim/sulfamethoxazole as prophylaxis for recurrent otitis media prior to the onset of symptomatic toxoplasmosis ^5^. Disseminated *T. gondii* infection with ocular manifestations has been reported in a patient with ataxia telangiectasia ^10^. This patient did not have lymphopenia and had received IVIg replacement therapy. In addition, fatal cerebral toxoplasmosis was reported in two patients with common variable immunodeficiency ^11, 12^.

Thus, hypogammaglobulinemia is a common feature of primary immunodeficiency disorders in which toxoplasmosis has been reported, suggesting the importance of antibody-mediated immunity for controlling *T. gondii*. Although our patient had a total IgG level just above the lower limit of normal, he developed a strong specific IgG response to *T. gondii*. The quality of the antibodies and the kinetics of the response are unknown but evidently were insufficient to initially control the pathogen. Cell-mediated immunity is also important in control of *T. gondii* infection, with critical complementary roles for monocytes, neutrophils, dendritic cells, plasma cells, and CD4+ and CD8+ T cells ^13^. IFNγ and IL-12 are hallmarks of the Th1 response to *T. gondii* infection ^13^. Neutrophils, activated monocytes, macrophages, and dendritic cells all produce IL-12, whereas IFNγ is produced by NK cells, neutrophils, and effector T cells in response to *T. gondii* invasion ^13^. In limited studies, mature DCs from WHIM patients have been reported to produce normal amounts of interleukin-12 (p70) and IFN-γ production has been reported to be similar between CD4 ^+^ T cells from a healthy donor and a WHIM patient activated by anti-CD3– and anti-CD28–coated beads ^14, 15^.

An explanation for the paucity of symptomatic *T. gondii* infections among patients with primary immunodeficiency is lacking. Possible explanations include the frequent use of broad-spectrum antibiotics such as trimethoprim-sulfamethoxazole for patients with primary and acquired immunodeficiency disorders and environmental precautions taken to limit exposure to pathogens in general.

WHIM syndrome is a complex, phenotypically heterogenous primary immunodeficiency disorder that frequently involves defects in steady state levels of leukocytes and antibody in the blood, as in our patient. Given that the patient has multiple immunologic abnormalities, it is not possible to attribute his susceptibility to *T. gondii* to any single one. A previous study has detailed a role for CXCR4 in regulating Plasmodium development in mouse and human hepatocytes, but no animal studies have been published to date that evaluate CXCR4 signaling in T. gondii or other protozoan infections ^16^.

In summary, we describe in detail a very rare case of primary ocular toxoplasmosis in primary immunodeficiency disease and the first case of protozoan infection in WHIM syndrome. The precise immunologic abnormalities among the spectrum of abnormalities resulting from WHIM syndrome that predisposed the patient to such a devastating outcome of *T. gondii* infection are currently unknown.

## Consent

Written informed pediatric assent was obtained from the patient, and the patient’s mother provided parental written informed consent for the publication of the patient’s clinical details and any associated images.

## Data availability

All data underlying the results are available as part of the article and no additional source data are required.
