# Supplementary material for: Case Report: Ocular toxoplasmosis in a WHIM syndrome immunodeficiency patient
Source: F1000Res. 2019 Jul 17;8:2. Originally published 2019 Jan 2. [Version 2] doi: 10.12688/f1000research.16825.2 (PMC6587139; doi:10.12688/f1000research.16825.2)
Supplement: Peer review history

# Peer review: Reviewer response for version 1

**DOI:** 10.5256/f1000research.18392.r49621

**Published:** 2019-07-10

**Authors:** Lynnette J. Mazur

**Affiliations:** 1 University of Texas Health Science Center at Houston, Houston, TX, USA

The Center for Disease Control notes that ‘parasitic infections are typically associated with poor and often marginalized communities in low-income countries. However, these infections are also present in the United States.’ Additionally, they have designated toxoplasmosis a neglected parasitic infection and a public health priority because of the little attention it has received ^1^. Based on the number of people infected, the severity of illness, and the ability for prevention and treatment, health care providers need to take note of its increasing prevalence along with the common signs and symptoms.  The case report by McDermott *et al.* provides this opportunity.   

 The report details a teen with a WHIM syndrome, a primary immunodeficiency disorder, who developed sudden painless right eye blindness due to ocular toxoplasmosis (OT) or toxoplasmic choroiditis ^2^. However, it is unknown whether his infection was congenitally acquired or acquired later in life through contaminated food or water or possibly as a result of a blood transfusion during his Tetralogy of Fallot repair. The maternal prenatal history, birth history, and the timing of his immigration might yield some clues.  There is evidence that persons with congenital infection have a higher incidence of macular involvement, as in this case, and therefore, have an increased risk of legal blindness ^3^.

 Additionally, testing for IgM and performing a quantitative IgG avidity test may help differentiate acute from chronic infection. The avidity test is based on the immovability antigen and antibody attachment where the IgG antibody binds weakly to the antigen during the initial stage but over weeks or months progressively increases ^4^ ^5^. Patients with positive IgM and/or low IgG avidity are considered as acutely acquired cases (usually defined as acquisition of infection in recent six months. Alternately, a negative IgM and a high IgG avidity index support a more chronic infection. A recent study on immune mediators in the aqueous humor showed that IL-9 and IL-13 were significantly higher in primary OT than in recurrent OT ^6^.

 Cases of isolated OT commonly results from reactivation of untreated congenital infection which can occur years after birth. However, it also occurs in a small percentage of people with acquired infection, especially in patients that are immunocompromised. This case heightens the awareness of the signs and symptoms, and consequences of toxoplasmosis infection for healthcare providers. It serves as an opportunity to educate patients, especially in international persons and immunocompromised individuals on common exposures, preventive measures, and treatment.

I confirm that I have read this submission and believe that I have an appropriate level of expertise to confirm that it is of an acceptable scientific standard.

## References

[1] : Neglected parasitic infections in the United States: toxoplasmosis. *Am J Trop Med Hyg*.2014;90(5) : 10.4269/ajtmh.13-0722 794-799 10.4269/ajtmh.13-0722 24808246PMC4015566

[2] : Case Report: Ocular toxoplasmosis in a WHIM syndrome immunodeficiency patient. *F1000Research*.2019;8: 10.12688/f1000research.16825.1 10.12688/f1000research.16825.1 PMC658713931249677

[3] : Ocular toxoplasmosis: clinical features and prognosis of 154 patients. *Ophthalmology*.2002;109(5) :869-78 1198609010.1016/s0161-6420(02)00990-9

[4] : Diagnosis of antigenic markers of acute toxoplasmosis by IgG avidity immunoblotting. *Parasite*.2013;20: 10.1051/parasite/2013017 18 10.1051/parasite/2013017 23688778PMC3718541

[5] : Serologic Tests of IgG and IgM Antibodies and IgG Avidity for Diagnosis of Ocular Toxoplasmosis. *Korean J Parasitol*.2018;56(2) : 10.3347/kjp.2018.56.2.147 147-152 10.3347/kjp.2018.56.2.147 29742869PMC5976017

[6] : Immune Mediator Profile in Aqueous Humor Differs in Patients with Primary Acquired Ocular Toxoplasmosis and Recurrent Acute Ocular Toxoplasmosis. *Mediators Inflamm*.2019;2019: 10.1155/2019/9356728 9356728 10.1155/2019/9356728 30906227PMC6398019

# Peer review: Reviewer response for version 1

**DOI:** 10.5256/f1000research.18392.r49539

**Published:** 2019-06-20

**Authors:** Françoise Bachelerie

**Affiliations:** 1 Inflammation Chemokines and Immunopathology, INSERM, Fac. de médecine—Univ Paris-Sud, Université Paris-Saclay, Clamart, France

I **s the background of the case’s history and progression described in sufficient detail?**

 David McDermott and colleagues are reporting the first example of protozoan infection ( *Toxoplasma gondii*) in the context of the WHIM syndrome. The patient displays the characteristic clinical features of the syndrome including Human Papillomavirus (HPV)-induced warts, Myelokathexis and severe panleukopenia as detailed in the manuscript. He harbors a well-known CXCR4 mutation (R334X) most likely inherited from one of his parents (although sporadic cases were reported) but this information is not provided. Along this line, whether parents or close relatives also present with serum IgG for T. *gondii *is likely but not provided. It is interesting to note that the patient developed dengue virus infection one year before being diagnosed for *Toxoplasma gondii *but was negative for serum IgG (witnessing a primary infection?).

**Are enough details provided of any physical examination and diagnostic tests, treatment given and outcomes?**

 Diagnostic for *Toxoplasma gondii* infection and treatment given are clearly mentioned. It would be also interesting that the authors mention about the evolution of *Toxoplasma gondii* infection by PCR testing during the 4 years follow up in the course of the daily trimethoprim/sulfamethoxazole treatment. The authors mention that infection was detected in vitreous fluid but not in serum nor in bone marrow. Were they other sites tested that would help to better understand the protozoan infection pathogenesis (acquisition/reactivation)? The authors mention that the patient successfully completed phase 3 plerixafor and filgrastim-based clinical trial. It will be thus beneficial for a better understanding of the patient treatment and outcome, to indicate when the patient was enrolled into this trial with regard to the *T. gondii* 4 year-treatment and, if appropriate, whether any follow up of the infection and responses toward it (e.g. serum Ig) was performed in the course of this trial.

 I **s sufficient discussion included of the importance of the findings and their relevance to future understanding of disease processes, diagnosis or treatment?**

 As emphasized by David McDermott and colleagues, protozoan infections are rare in primary immunodeficiency disorders and this is first example of such infection in the context of the WHIM syndrome. Authors provide interesting hypotheses that might account for the clinical manifestations of *Toxoplasma gondii* infection and pathogenic mechanisms. I would recommend them to add recent literature regarding the potential role of CXCR4 in the life cycle of other members of the phylum Apicomplexa ( *Plasmodium) *assessed in rodent models ^1^ *.* For other parasites such as helminths ( *e.g.* L. sigmodontis) analyzed in rodent models of infection, neutrophils (and their control by a CXCR4/CXCL12 dependent mechanism) were proposed to be critical elements of the host innate protective response ^2^.

 I **s the case presented with sufficient detail to be useful for other practitioners?**

 The report of this singular case is of great interest for scientists and clinicians working in the field and certainly very useful for practitioners not well aware of the syndrome.

I confirm that I have read this submission and believe that I have an appropriate level of expertise to confirm that it is of an acceptable scientific standard.

## References

[1] : CXCR4 regulates Plasmodium development in mouse and human hepatocytes. *J Exp Med*.2019; 10.1084/jem.20182227 10.1084/jem.20182227 31189656PMC6683982

[2] : Neutropenic Mice Provide Insight into the Role of Skin-Infiltrating Neutrophils in the Host Protective Immunity against Filarial Infective Larvae. *PLoS Negl Trop Dis*.2016;10(4) : 10.1371/journal.pntd.0004605 e0004605 10.1371/journal.pntd.0004605 27111140PMC4844152

# Peer review: Reviewer response for version 1

**DOI:** 10.5256/f1000research.18392.r42400

**Published:** 2019-01-21

**Authors:** Sarah Beaussant-Cohen

**Affiliations:** 1 Division of Immunology, Boston Children’s Hospital, Harvard Medical School, Boston, MA, USA

**Question 1: Is the background of the case’s history and progression described in sufficient detail?**

 David H. McDermott *et al* report the first advanced ocular toxoplasmosis complication in WHIM syndrome. The case is interesting because the patient exemplifies widely known aspects of the disease, demonstrating classic features of WHIM syndrome, while at the same time manifests aspects of the syndrome which clinicians unfamiliar with the disease may not be aware of such as biological combined immune deficiency or a history of Tetralogy of Fallot (Raffaele Badolato, J Pediatr. 2012) ^1^.

 Importantly, this case exemplifies that manifestations reported in the acronym “W.H.I.M” (Warts, Hypogammaglobulinemia, Infections and Myelokathexis) insufficiently describe the disease and may even be misleading. Indeed, while the patient clearly presents three of the manifestations emphasized in the acronym 1) Warts 2) a history of recurrent upper and lower respiratory tract Infections and 3) Myelokathexis, the patient does not show biologically defined hypogammaglobulinemia. However, while his IgG levels are in the normal range (according to Table 1), his clinical presentation with repeated respiratory and ENT infections are typical of patients with hypogammaglobulinemia. Furthermore, in the discussion section, the authors suggest that the patient appears to have a qualitative humoral defect. This case clearly demonstrates that the spectrum of WHIM syndrome manifestations range well-beyond the acronym. Importantly, the reader will appreciate that the patient’s labs are compatible with combined immunodeficiency: CD4+ T cells 365, CD19+ B cells 11 [per microliter] and very low naive CD4+ and CD8+ T cells.

 We can conclude that the authors have given an accurate description of a typical WHIM patient ( *CXCR4R334X* ) who presents not only well-known manifestations of the disease (neutropenia, warts) as well as lesser known (Tetralogy of Fallot, combined immune deficiency) hallmarks of the disease.  This detailed report will greatly help clinicians better apprehend and recognize this complex syndrome in their own patient population.

**Question 2: Are enough details provided of any physical examination and diagnostic tests, treatment given and outcomes?**

 The authors describe in detail the challenging diagnosis of toxoplasmic optic neuritis. As a reader, we understand the medical thought-process which led to the diagnosis.  Due to the neutropenia, the patient did not initially show strong clinical findings of ocular toxoplasmosis. However, Filgrastim administration unmasked vitritis which is a prominent feature of ocular toxoplasmosis. The diagnosis was then confirmed by PCR testing which was positive for *T. gondii *in vitreous fluid. The patient received standard management of toxoplasmosis-associated optic neuropathy *as *he was treated with oral pyrimethamine (75 mg loading dose then 25 mg/day), leucovorin (7.5 mg/day), and sulfadiazine (1500 mg 2x/day) for six weeks (Rim Kahloun *et al*, Eye Brain. 2015) ^2^. Optic neuritis remains an unusual presentation of ocular Toxoplasmosis that is associated with worse visual outcome. This is clearly shown during the prolonged follow-up of the patient who sustains findings of fibrotic and atrophic macula without signs of active exudative lesions.

**Question 3: Is sufficient discussion included of the importance of the findings and their relevance to future understanding of disease processes, diagnosis or treatment?**

 The authors are well aware that rare monogenic diseases such as WHIM syndrome offer a unique window of insight into the role of the CXCR4 pathway in health and disease. In their discussion, the authors offer a brief explanation of the possible pathophysiology of the appearance of toxoplasmic optic neuritis in this WHIM patient. They suggest that he presents with both impaired humoral immunity and defective cell-mediated immunity. In particular, they address the question of a defective IFN-gamma and/or IL-12 pathway in this disease. As of today, there is not a sufficient amount of work in the literature to address this question properly, nevertheless it may have been interesting to cite previous studies such as the work of Laura Tassone *et al *(Blood 2010) ^3^which suggested that mature DCs from WHIM patients produce normal amounts of interleukin-12 (p70) compared with the cells derived from healthy donors or the work of Marinos Kallikourdis *et al *(blood 2013- figure 5c) ^4^ in which she shows that IFN-gamma production is not different between CD4 ^+^ T cells from a healthy donor or a WHIM patient (G336X) activated by anti-CD3– and anti-CD28–coated beads. Clearly, the questions raised by the authors are pertinent and underline the importance of further studies to better characterize the defects in this pathway.

**Question 4: Is the case presented with sufficient detail to be useful for other practitioners?**

 WHIM is often classified as a severe congenital neutropenia, which may be misleading to clinicians unfamiliar with the disease.  Therefore, this case will be very useful to clinicians involved in the care of patients with WHIM syndrome. This report will remind clinicians that they should keep a high degree of suspicion at all times as it is possible that their WHIM patients may present with unusual infections classically seen in patients with severe T cell immunodeficiency.

**Minor Changes to be addressed:**

 Table 1:

- lines for both NK and NK-T are duplicated.
- Patient does not have IgG hypogammaglobulinemia according to reference ranges in table 1. This should be stated in the text.
- It could be useful to bold the values with are outside of the normal range in table 1.

I confirm that I have read this submission and believe that I have an appropriate level of expertise to confirm that it is of an acceptable scientific standard.

## References

[1] : Tetralogy of fallot is an uncommon manifestation of warts, hypogammaglobulinemia, infections, and myelokathexis syndrome. *J Pediatr*.2012;161(4) : 10.1016/j.jpeds.2012.05.058 763-5 10.1016/j.jpeds.2012.05.058 22748845PMC3458406

[2] : Infectious optic neuropathies: a clinical update. *Eye Brain*.2015;7: 10.2147/EB.S69173 59-81 10.2147/EB.S69173 28539795PMC5398737

[3] : Defect of plasmacytoid dendritic cells in warts, hypogammaglobulinemia, infections, myelokathexis (WHIM) syndrome patients. *Blood*.2010;116(23) : 10.1182/blood-2010-03-272096 4870-3 10.1182/blood-2010-03-272096 20736454

[4] : The CXCR4 mutations in WHIM syndrome impair the stability of the T-cell immunologic synapse. *Blood*.2013;122(5) : 10.1182/blood-2012-10-461830 666-73 10.1182/blood-2012-10-461830 23794067PMC3731928
